# Social Connections and Community Contexts: Shaping End-of-Life Plans Among U.S. Older Adults

**DOI:** 10.1093/geroni/igaf122.146

**Published:** 2025-12-31

**Authors:** Deborah Carr, Lucie Kalousova, Clifford Ross

## Abstract

Advance care planning (ACP) is an important step toward ensuring individuals’ end-of-life (EOL) wishes are honored. Yet, many U.S. older adults do not engage in these important practices. This symposium presents four original studies that explore how relationship histories, family dynamics, social connections, and neighborhood environments affect older adults’ ACP behaviors, drawing on recent data from the Health and Retirement Study (HRS). Carr and Choi examine how complex romantic partnership histories and parental status affect four subtypes of ACP (formal advance directives only. informal discussions only, both formal and informal, or neither), revealing that recently widowed individuals are the most likely to engage in ACP, whereas childless persons and those who have never married are notably less involved. Zhang and Kalousová delve into the role of complex relationship histories, demonstrating that marital disruptions and remarriage significantly impact ACP decisions, particularly around establishing living wills and assigning durable power of attorney for healthcare (DPAHC). Lian and Kalousová explore how friendship networks influence ACP, uncovering important gender differences and emphasizing the critical role of friend-based emotional support for never-married older men. Lastly, Ding, Lou, and Carr highlight how neighborhood characteristics, such as cohesion and disorder, influence ACP participation among older adults living alone, particularly enhancing ACP engagement for women living in cohesive neighborhoods. Collectively, these studies provide valuable insights into the social and relational influences on ACP, informing strategies and policies aimed at improving end-of-life preparedness across diverse groups of older adults.

